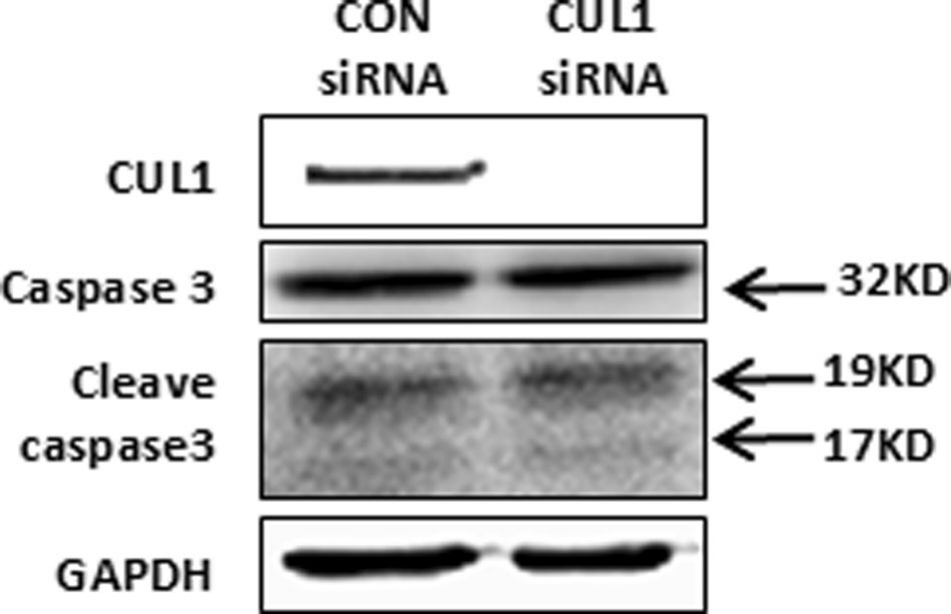# Correction: CUL1 promotes trophoblast cell invasion at the maternal–fetal interface

**DOI:** 10.1038/s41419-025-07994-2

**Published:** 2025-09-30

**Authors:** Q. Zhang, Q. Chen, X. Lu, Z. Zhou, H. Zhang, H-Y Lin, E. Duan, C. Zhu, Y. Tan, H. Wang

**Affiliations:** 1 State Key Laboratory of Reproductive Biology, Institute of Zoology, Chinese Academy of Sciences, Beijing, China; 2 Laboratory Animal Center, Chongqing Medical University, Chongqing, China; 3 Graduate School of Chinese Academy of Sciences, Beijing, China; 4 Department of Obstetrics and Gynecology, Beijing Obstetrics and Gynecology Hospital, Capital Medical University, Beijing, China

Correction to: *Cell Death and Disease* 10.1038/cddis.2013.1, published online 21 February 2013

Due to an oversight regarding image usage in published article, two identical images of interference with CUL1 expression bands in Figure 4b and Figure 5c were mistakenly included in the manuscript. We are fully prepared to provide revised figures or any additional materials required to rectify this error and ensure the accuracy of the published content.

**Incorrect Figure** 
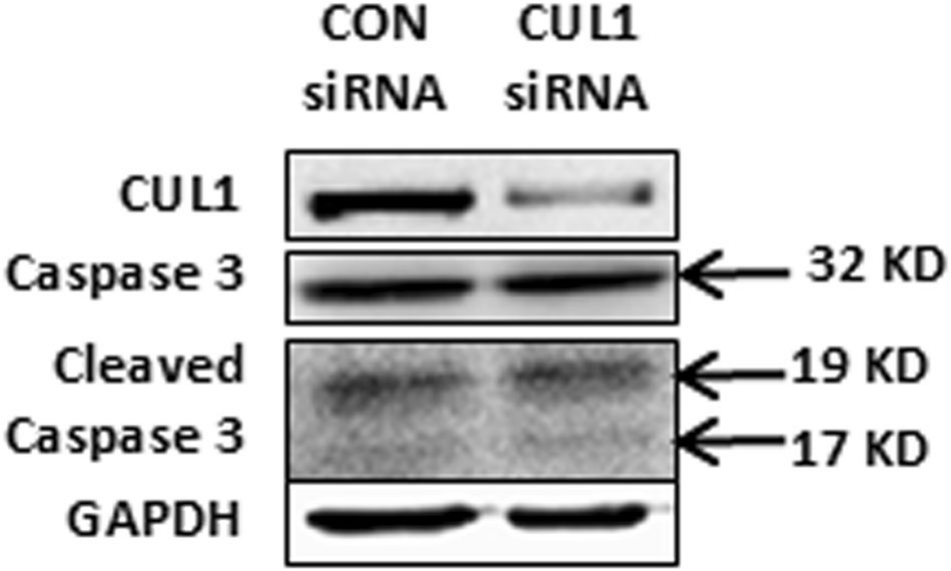


**Correct Figure**